# Supplementary material for: Study of Bayesian variable selection method on mixed linear regression models
Source: PLoS One. 2023 Mar 17;18(3):e0283100. doi: 10.1371/journal.pone.0283100 (PMC10022788; doi:10.1371/journal.pone.0283100)
Supplement: Attachment — Submitted filename: Report on PONE-D-22-18597_reviewer.pdf [file pone.0283100.s002.pdf]

Referee report on *PLOS one* submission: "Study of Bayesian variable selection method on linear mixed regression models"

Manuscript ID: PONE-D-22-18597

General comments:

The submitted manuscript investigates the Bayesian variable selection problem in the context of linear mixed model with implicit state. The LASSO-type penalty term is employed as variable selection instrument and a Gibbs-type algorithm based on Laplacian-prior is proposed to implement the method. Some numerical studies are conducted to illustrate the performance of the proposed variable selection instrument. Variable selection (or more generally the model selection) is always the first problem needs to be answered in data analysis and investigating the Bayesian variable selection in the domain of linear mixed models is an important issue. However, after reading the manuscript, I feel that the research problem has not been investigated thoroughly. In specific, I have some concerns on the hyperparameter selection, design of simulation study and also the case study. Please see my specific comments below.

Specific comments:

1. Bayesian variable selection provides a promising way to interpret the underlying mechanism of LASSO method. However, the Bayesian framework also induces further difficulties and one of the most important issues is how to determine the hyperparameters. In current study, very unfortunately, the authors failed to provide any details on how to determine hyperparameters in their proposed method (they just simply mentioned the value of hyperparameters used in the simulation). There are a number of immediate problems. Whether they are prefixed constants satisfy certain conditions? If so, any theoretical reasons? Or being selected from a set of candidate values based on a data-driven criterion? How to avoid overtraining of overfitting? I want to see some in-depth theoretical and numerical investigations about these issues in the revised version.

2. In principle, there are two typical settings for model selection, i.e. the "true-model world" where the trued data generating process (DGP) is nested within the candidate model set, and the "non-true model world" where DGP are unknown (Flynn et al., 2013). The current

manuscript studies the first setting. In linear models, Flynn et al. (2013) show that LASSO provides efficient model selection results. Then, another immediate question is that in the "non-true model world", whether the proposed model selection instrument can also possess similar properties?

3. In the simulation study, the authors imposed a very strong signal-to-noise setting, where non-zero coefficients are large in general. I'd like to see some investigations on relative weak signal settings.

4. The case study did not provide too much information! Is there any scientific evidence showing that the identified variables are reasonable or not? Whether the selected model yields good prediction performance or not? Please consider this carefully!

Minor comments:

1. Tables are separated on different pages and you can simply use floating tables to avoid this.

References

Flynn, C.J., Hurvich, C.M. and Simonoff, J.F., (2013) Efficiency for Regularization Parameter Selection in Penalized Likelihood Estimation of Misspecified Models *Journal of the American Statistical Association*, **108**, 1031—1043.
