# Supplementary material for: Study of Bayesian variable selection method on mixed linear regression models
Source: PLoS One. 2023 Mar 17;18(3):e0283100. doi: 10.1371/journal.pone.0283100 (PMC10022788; doi:10.1371/journal.pone.0283100)
Supplement: Attachment — Submitted filename: Review report of PONE-D-22-18597.pdf [file pone.0283100.s003.pdf]

Review report of “Study of Bayesian variable selection method on linear mixed regression models”

This paper introduces the Bayesian adaptive group Lasso method to investigate variable selection for the mixed linear regression model with an implicit state and explanatory variables with a grouping structure. The topic is interesting and the paper is well-written.

Comments:

1. Page 5, lines 5-6: Why is $[y_{it}|S_{it} = s]$ the observed time? What does the brackets mean?
2. An important aspect of any Bayesian analysis is when we use (close to or) noninformative priors, posterior distributions can be improper (see, Hobert and Casella, 1996). Hence, it is also important to have a complete sensitivity study about the choice of the hyper-parameters for the priors.
3. For reproducibility purposes, I would suggest to make the code available (if it is possible) either as supplementary material to be published online or as a reference to a gitHub website.

Minor Comments:

1. Pages 9-10: It seems unnecessary to replace $(\theta, \sigma^{-2}, \cdots)$ with $(\zeta_1, \cdots, \zeta_6)$.
2. For comparison purpose, could you combine Table 1-3 and redesign the table?

References

[1] Hobert, J. P., Casella, G. (1996). The effect of improper priors on Gibbs sampling in hierarchical linear mixed models. *Journal of the American Statistical Association*, **91**(436), 1461-1473.
