# Supplementary material for: Study of Bayesian variable selection method on mixed linear regression models
Source: PLoS One. 2023 Mar 17;18(3):e0283100. doi: 10.1371/journal.pone.0283100 (PMC10022788; doi:10.1371/journal.pone.0283100)
Supplement: Attachment — Submitted filename: Response to Reviewers.pdf [file pone.0283100.s004.pdf]

RE: "Study of Bayesian variable selection method on mixed linear regression models"

We are very grateful to the Editor, Associate Editor, and two reviewers for their constructive comments and suggestions, which have helped greatly in improving our paper. Our point-by-point responses are given below.

Response to Reviewer 1

1. *Bayesian variable selction provides a promising way to inerpret the underlying mechanism of LASSO method. However, the Bayesian framework also induces further difficulties and one of the most important issues is how to determine the hyperparameters. In current study, very unfortunately, the authors failed to provide any details on how to determine hyperparameters in their proposed method (they just simply mentioned the value of hyperparameters used in the simulation). There are a number of immediate problems. Whether they are prefixed constants satisfy certain conditions? If so, any theoretical reasons? Or being selected from a set of candidate values based on a datadriven criterion? How to avoid overtraining of overfitting? I want to see some in-depth theoretical and numerical investigations about these issues in the revised version.*

   Response: We are very grateful to the reviewer for the valuable comments. In order to observe the influence of the value of the hyperparameter on the model inference, we added the hyperparameter sensitivity test, and the experimental results of the two groups of hyperparameters were close.This shows that the inference method proposed in this paper is insensitive to hyperparameters. Therefore, it is only necessary to select some non-information priors when setting the hyperparameters of prior distribution. Please refer to page 11 of the revised version.

2. *In pinciple, there are two typical setings for model selection i.e. the "true -model worldr" where the trued data generating process (DGP)is nested within the candidate model set, and the "non-true model world where DGP are unknown (Fynn et al, 2013) The curent manuscript studies the first setting. In linear models, Flynn et al. (2013) show that LASSO provides efficient model selection results. Then, another immediate question is that in the "non-true model world", whether the proposed model selection instrument can also possess similar properties?*

   Response:Thank you for the insightful point.You are right.We studied the first setting.To be honest,we haven't considered the second case.After you asked this question,we had some thoughts. Maybe we will do some word in this field in the future .However,due to the immature consideration,we dare not answer this question randomly.Please forgive us.

3. *In the simulation study, the authors imposed a very strong signal-to-noise setting, where non-zero coefite are large in general. l'd like to see some ievetgaions on relaive weak signal settings.*

   Response: Thank you for your valuable question. We have reduced the non-zero coefficient and resimulation experiment according to your suggestion. Please refer to page 8 of the revised version.

4. *The case study did not provide too much information! Is there any scientific evidence showing that the identifed variables are reasonable or not? Whether the selected model yields good prediction performance or not? Please consider this carefull!*

   Response:We are very greatful to the reviewer for the valuable comment. We use the method to analysis a new Alzheimer's disease data. With the increase of humen life expectancy, there are more and more patients with Alzheimer's disease,so the research on Alzheimer's disease is more and more important. Varible selection can screan out the factor that may affect Alzheimer's disease, which is vary meaningful. We studied the Alzheimer's disease dataset with the proposed model and method, and analyzed the results of vasiable selection and parameter estimation. Please refer to page 12 of the revised version.

5. *Tables are separated on differrent pages and you can simply use floating tables to avoid this.*

   Response:Thank you for raising the good point. We deleted Table 2 and redesigned the table to merge Table 3 with Table 1. Please refer to page 9 of the revised version.

Response to Reviewer 2

1. *Page 5, lines 5-6: Why is* $[y_{it}|S_{it} = s]$*the observed time? What does the brackets meau?*

   Response: Sorry, we left out something in the original text. The revised paper has added "observe time is $t = 1, 2, \cdots T$". In the original paper, we want to use the brackets to represent the conditional distribution, that is, the y distribution of the observing variables in the state of s. The revised paper uses a more rigorous expression. Please refer to page 3 of the revised version.

2. *An important aspect of any Bayesian analysis is when we use (close to or) noninformative priors, posterior distributions can be improper (see, Hobert and Casella, 1996). Hence, it is also important to have a complete sensitivity study about the choice of the hyper-parameters for the priors.*

Response: We are very grateful to the reviewer for the valuable comments.In order to observe the influence of the value of the hyperparameter on the model inference, we added the hyperparameter sensitivity test, and the experimental results of the two groups of hyperparameters were close.This shows that the inference method proposed in this paper is insensitive to hyperparameters. Therefore, it is only necessary to select some non-information priors when setting the superparameters of prior distribution.Please refer to page 11 of the revised version.

3. *For reproducibility purposes, I would suggest to make the code available (if it is possible) either as supplementary material to be published online or as a reference to a gitHub website.*

   Response:Thanks for the reviewer's suggestion! We uploaded all the R codes for the experiment as you suggested.

4. *Pages 9-10: It scems unnecessary to replace* $(\theta, \sigma^{-2}, \cdots)$*with*$(\zeta_1, \cdots, \zeta_6)$.

   Response:Thank you for the insightful point. Since the $(\theta, \sigma^{-2}, \cdots)$with superscripts and subscripts will be confused with the labels in the MCMC algorithm, we replace them with$(\zeta_1, \cdots, \zeta_6)$, which will make the MCMC algorithm clearer.

5. *For comparison purpose, could you combine Table 1-3 and redesign the table?*

   Response: We are very grateful to the reviewer for the valuable comment. We deleted Table 2 and redesigned the table to merge Table 3 with Table 1. Please refer to page 9 of the revised version.
