# Supplementary material for: Study of Bayesian variable selection method on mixed linear regression models
Source: PLoS One. 2023 Mar 17;18(3):e0283100. doi: 10.1371/journal.pone.0283100 (PMC10022788; doi:10.1371/journal.pone.0283100)
Supplement: Attachment — Submitted filename: Response to Reviewers.pdf [file pone.0283100.s005.pdf]

**RE: "Study of Bayesian variable selection method on mixed linear regression models"**

We are very grateful to the Editor, Associate Editor, and two reviewers for their constructive comments and suggestions, which have helped greatly in improving our paper. Our point-by-point responses are given below.

**Response to Reviewer 1**

1. *1. Many mathematical notations are not displayed appropriately, e.g. "expa" should be "*exp*" in Equations (5)—(8); there are typos in Equation (8) and so on. These problems severely affect the readability of the paper. Can you employ LaTex or other professional software to handle mathematical notations?*

   Response: Thank you for your question! We have made modifications according to your suggestions. Please refer to page 5 of the revised version.

2. *There are some language issues, e.g., "Case analysis" should be "Case study", "interest covariates" should be "covariates of interest" and so on. Proof read is needed!*

   Response:We are very grateful to the reviewer for the valuable comments. We have carefully proofread and revised the article. We have revised many places and marked them in red in the article.

3. *Please describe how did you select 512 patients from the original data set.*

   Response: Thank you for your valuable question. We have eliminated the individuals with missing information, and the remaining 512 individuals. Please refer to page 12 of the revised version.
